# Etiological characterization of acute poisonings in the emergency department

**DOI:** 10.4103/0974-2700.50878

**Published:** 2009

**Authors:** Malek Khlifi, Leslie Zun, Giffe Johnson, Raymond Harbison

**Affiliations:** 1 Center for Environmental/Occupational Risk Analysis and Management, Department of Environmental and Occupational Health, College of Public Health, University of South Florida, Tampa, Florida 33612, USA; 2 Mount Sinai Hospital, Emergency Department, California Avenue at 15^th^ Street, Chicago, IL 60608, USA

**Keywords:** Poisonings, etiology, emergency medicine

## Abstract

**Introduction:**

An investigation of emergency department (ED) poisonings was conducted to characterize poisoning demographics and evaluate correlations with select co-morbidities.

**Methods:**

The study population evaluated consisted of 649 poisoning cases admitted between 2004 and 2007 to an inner-city, level 1 emergency department.

**Results:**

Ethnicity, age, and gender had a substantial impact on the population distribution as poisoning cases were predominantly African Americans (79.9%) between 36 and 45 years old with a 1:3 male to female ratio. Intentional illicit drug overdose was the most prevalent cause of poisoning, heroin being the most frequent substance found in 35.4% (n = 230) of cases, followed by cocaine overdose at 31.7% (n = 206), concomitant heroin and cocaine overdose at 4.3% (n = 28), multiple drug poisoning at 5.5% (n = 36), and antidepressant/antipsychotic poisoning at 6% (n = 39). Significant correlations were found between heroin poisoning and asthma (F = 20.29, DF = 1, *P* = 0.0001), cocaine poisoning and hypertension (F = 33.34, DF = 1, *P* = 0.0001), and cocaine poisoning and cardiovascular disease (F = 35.34, DF = 1, *P* = 0.0001). A change in the pattern of illicit drug use from injection to inhalation was detected and the resulting increase of inhalation and insufflation of illicit substances may partially explain the correlation found between heroin use and asthma.

**Conclusions:**

These results provide supporting evidence that deliberate poisoning with illicit drugs remains a serious healthcare issue that significantly aggravates co-morbidities and raises treatment costs by increasing both the rate of hospitalization and hospital length of stay.

## INTRODUCTION

Poisonings from substance abuse and accidental toxic exposure remain a significant concern for hospital emergency departments (ED), with more than four million incidents of poisoning occurring in the United States each year. The Institute of Medicine has recently identified poisoning as the second leading cause of injury-related mortality, with an estimated health care cost of over $12.6 billion annually.[1] Over 86 percent of all reported poisoning cases were related to accidental exposure, while suicidal or self poisoning accounted for less than 8% of total poisonings. However, emergency department visits for attempted suicide and self-inflicted injury are relatively common and often have a poor prognosis for full recovery. Of these cases, self-poisoning is the most common method for both attempted and successful suicides.[2,3]

Substance abusers are frequently seen in the ED and the predominating substances being abused generally reflect regional and local trends for illicit drug use. Trends in the United States for illicit drug use indicate that cocaine abuse decreased between 1992 and 2002, though patterns of abuse were not consistent throughout the country as cocaine use actually increased 100% or more in four states during this same time period.[4] Among persons aged 12 or older, the rate of current illicit drug use in 2007 was 9.3 percent in the West, 7.9 percent in the Midwest, 7.8 percent in the Northeast, and 7.4 percent in the South. Current marijuana use declined from 2002 to 2007 in each region, although the difference was not significant in the West. In 2007, the rate of current illicit drug use was higher in metropolitan areas than in nonmetropolitan areas. The rates were 8.3 percent in large metropolitan counties, 8.2 percent in small metropolitan counties, and 6.7 percent in nonmetropolitan counties as a group. Within nonmetropolitan areas, the rate was 7.5 percent in urbanized counties, 6.7 percent in less urbanized counties, and 4.1 percent in completely rural counties. The rate of current illicit drug use among the population aged 12 or older in completely rural counties in 2007 (4.1 percent) was lower than that observed in 2006 (7.8 percent).[5]

Heroin use remains a serious problem in some regions of the United States. A recent change in the route of heroin use has been observed, from a decrease in intravenous injection to the practice of heating heroin in aluminum foil for inhalation. There has also been an increasing trend for mixing heroin with cocaine to be smoked in this manner.[6]

Although heroin and cocaine remain the most frequently abused drugs leading to ED visits, ED visits involving prescription narcotic analgesics increased 153% from 1995 to 2002. Concomitant use of drugs were involved in 75% of the drug abuse–related ED visits, and dependence was an underlying factor in these cases. Substance abusers are more likely to be hospitalized for acute intoxication, and 2.3 times more likely to use an emergency room than non-abusers.[6,7]

The association of drug abuse and respiratory co-morbidities, particularly between cocaine and asthma, has been reported sporadically in the scientific literature. In an investigation conducted in Bronx, NY, a correlation between cocaine use and new onset asthma was reported amongst substance abusers.[8] Steensen *et al*. 1993 reported a relationship between heroin abuse and pulmonary edema, which was supported by Marby *et al*. 2004 who found a pattern of heroin overdose-induced pulmonary edema.[9,10] Levine *et al*. 2005 found that cocaine and heroin abuse increased the rate of intubations and hospital utilization in patients with acute asthma exacerbation.[11]

Knowledge of condition-specific morbidity and mortality for patients with asthma as well as other aggravating factors is essential for making critical decisions in emergency care. Some evidence exists indicating that drug abuse may be increased among asthma patients and that drug use may exacerbate their condition, though the etiology and mechanism of action for this pathology has not been explained. There is a need, especially for emergency care providers in underserved minority communities with high frequencies of poisonings and substance abuse, to understand the demographic factors involved in poisoning and related co-morbidities.

## MATERIALS AND METHODS

The study population consists of Chicago city residents visiting the ED of the Sinai Hospital between December 2004 and May 2007. The Sinai Health System provides virtually all-medical and surgical care in the area, including a children's hospital and a rehabilitation center. The ED serves residents up to 5 miles west of the downtown area.

Data collection was performed according to HIPAA regulations after approval by the hospital's Institutional Review Board (IRB) committee. The setting was the ED of an inner city level one trauma center with approximately 45,000 visits per year. Patients between 1 and 89 years old and exposed to poisons counting chemical, recreational, and/or pharmaceutical agents were selected. The study population consisted of 649 poisoning cases admitted between 2004 and 2007 to a level 1 ED. Cases were identified using the International Classification of Disease Ninth Revision, (ICD-9-CM). Demographic health data abstracted from patients' medical records included age, gender, race, date and time of the visit, marital status, employment, insurance coverage, chief complaint, and vitals on admission. Data on personal medical history of diabetes mellitus, hypertension, cardio-vascular diseases, asthma, arthritis, HIV status, hepatitis, and seizure disorder was collected. The poisoning data collected referred to the type of poison, the poisoning exposure route, and the circumstances of intoxication (intentional or unintentional). The initial management of the event was also determined in an attempt to correlate it to the final disposition of the cases. Smoking, alcohol use, illicit drug use, insurance coverage, hospital charges, length of stay for those hospitalized, and any history of mental illness such as depression, psychosis, and anxiety disorder was collected from all case records.

Poisoning exposure was grouped into fifteen toxic substances or drugs in four separate categories: Pharmaceutical or medicinal drug use, recreational drug use, chemical exposure, and industrial exposure. Circumstances of the exposure were also captured and categorized into intent groups that included: Suicidal, abuse, misuse, unintentional exposure, therapeutic use, and adverse drug events (ADE). The route of exposure was recorded as: Inhalation, ingestion, injection, dermal, and rectal/vaginal. To assess poisoning burden inpatient length of stay (LOS) and health care expenditures on asthmatic patients with a history of drug addiction and poisoning were compared to those with asthma and addiction but with no history of poisoning.

Continuous variables such as age, length of hospital stay, and duration of time between poisoning and hospital arrival was analyzed using student's *t*-test or one-way analysis of variance (ANOVA) between groups. The categorical data between groups was analyzed with Chi square tests and full factorial univariate analyses by stepwise multiple logistic regression. Correlation analysis was conducted using the Pearson r correlation coefficient. A *P* value of less than 0.05 is considered statistically significant. The data was analyzed using SPSS 16.0 statistical package.

## RESULTS

Between December 2004 and May 2007 a total of 649 ED poisonings were identified. The majority of poisoning cases were between 36 and 45 years old. Pediatric cases younger than 15 years old represented only 4.6% of cases and the most frequently affected age was 48. Of the total of 649 poisoning cases there were more male, than female, with 42.7% (*n* = 277) female and 55.9% (*n* = 363) male. Poisoning cases were predominantly African Americans (79.9%) followed by Hispanics (11.9%), and Whites (6.6%). Over 69.3% of the total cases were single in comparison to 12.3% who were married. Most patients were uninsured (45.8%), while 35.1% were covered by the Illinois Department of Public Aid (IDPA), Medicare and Medicaid covered 8.2%, and only 5.9% of patients had private coverage.

The exposure substances identified as most commonly encountered in ED included: Heroin 35.4% (*n* = 230), cocaine 31.7% (*n* = 206), heroin and cocaine concomitantly taken 4.3% (*n* = 28), alcohol 2.2% (*n* = 14), multiple drugs 5.5% (*n* = 36), antidepressant/antipsychotic 6% (*n* = 39), ASA 3.4% (*n* = 22), cardio vascular drugs 2% (*n* = 13), anti-cold medications 1.1% (*n* = 2.2), methadone 0.6% (*n* = 4), CNS stimulants 0.8% (*n* = 5), Analgesics 1.5% (*n* = 10), Marijuana 0.5% (*n* = 3), unknown pills 1.7% (*n* = 11), and “other” substances 2.9% (*n* = 19). The routes of poisoning exposure included inhalation 61.5% (*n* = 400), ingestion 21.1% (*n* = 137), rectal/vaginal 10.6% (*n* = 69), and injection at 5.2% (*n* = 34.). The exposure circumstances included: Abuse 76% (*n* = 498), suicidal 13.4% (*n* = 87), misuse 2.2% (*n* = 14), unintentional 3.2% (*n* = 21), therapeutic 2.5% (*n* = 16), and ADE 0.6% (*n* = 4).

A significant relationship was found between asthma and the exposure to heroin (F = 20.4, DF = 1, *P* = 0.0001), as well as between history of cardio-vascular disease and exposure to cocaine (F = 35.34, DF = 1, *P* = 0.0001). Another significant relationship was detected for the use of cocaine and a history of preexisting hypertension (F = 33.34, DF = 1, *P* = 0.0001). Depression and psychosis were significantly associated with overall poisonings (F = 42.59, DF = 1, *P* = 0.0001).

A statistically significant relationship was found between poisoning exposure and the history of smoking (F = 2.90, DF = 14, *P* = 0.0001). Another significant relationship (F = 3.76, DF = 14, *P* = 0.0001) was detected between those with a history of alcohol use and poisoning. History of recreational drug use was also found to be significantly related (F = 33.64, DF = 14, *P* = 0.0001) to poisoning.

Over 31% (*n* = 199) of cases were diagnosed with a prior history of hypertension.

Among these, 27.1% (*n* = 54) used heroin and 47.2% (*n* = 94) used cocaine, 4% (*n* = 8) had used a combination of both heroin and cocaine, and 5.5% (*n* = 11) who used anti-depressants/anti-psychotics. Sixty percent (*n* = 121) reported they had taken other prescription medications on the day of their poisoning. Over 71.9% (*n* = 143) of patients with hypertension were hospitalized and only 26% were discharged from ED. Hypertension was not correlated to poisoning overall, however, it was correlated specifically to cocaine poisoning (*P* = 0.0001).

Depression among asthma patients was prevalent at 18.2%, and 8.6% (*n* = 23) were diagnosed and treated for a psychotic disorder. Most asthma patients had a history of recreational drug use 86.1% (*n* = 229). Therapeutic adverse events were also reported among asthma patients at a rate of 2.2% (*n* = 6) and misuse of drugs at a rate of 1.1% (*n* = 2).

A history of prior poisoning that led to an ED visit or hospitalization for similar events was present in 54.3% (*n* = 146) of asthmatics. Inhalation was the most commonly reported mode of exposure of asthmatics at 75.5% (*n* = 203), ingestion was seen in 11.5% (*n* = 31), then injection 6.3% (*n* = 17), rectal or vaginal at 4.5% (*n* = 12), and dermatological 1.1% (*n* = 3). Smoking was prevalent among asthmatics at 65.1% (*n* = 175) and regular alcohol intake was seen in 54.6% (*n* = 147) of asthmatics. At the time of the exposure, 67.7% of asthma patients reported that they were taking their asthma routine maintenance medication and 53.2% of the total asthma groups were under albuterol treatment.

A significant relationship between exposure to heroin and history of Asthma was found (*P* = 0.0001). Asthma was not correlated to any of the other substances of exposure including cocaine, alcohol, or concomitant use of heroin and cocaine. ANOVA resulted in a statistically significant relationship between asthma and depression (*P* = 0.044) and to psychosis (*P* = 0.028), however smoking was not statistically significantly associated with asthma with a *P* value slightly greater than 0.05 (*P* = 0.066).

The Pearson correlation test displayed a statistically significant relationship between asthma and heroin use (r = 0.175, DF = 647, *P* = 0.001). Heroin was also found to be correlated to many other variables in our population, such as depression, psychosis, and cardio-vascular diseases [Table 1].

**Table 1:** Summary of heroin poisoning correlations

Variable 1	Variable 2	R	DF	*P* value
Heroin poisoning	Vaginal or rectal route	0.350	645	0.0001
	History of drug abuse	0.286	635	0.0001
	Heart related CC	0.268	645	0.0001
	History illicit drug abuse	0.324	645	0.0001
	SOB (CC)	0.117	645	0.0001
	Asthma	0.174	645	0.0001
	Depression	−0.118	644	0.0001
	Suicidal poisoning	−0.234	645	0.0001

CC: CHIEF COMPLAINT SOB: SHORTNESS OF BREATH

Cocaine was found to be significantly correlated to chronic hypertension (r = 0.220, DF = 745, *P* = 0.0001) and to cardiovascular diseases (r = 0.227, DF = 645, *P* = 0.0001). Cocaine was also significantly correlated to heroin, to heroin and cocaine combined use, to multiple drug use, smoking, and to regular alcohol intake [Table 2].

**Table 2:** Significant correlations with cocaine poisoning

Variable 1	Variable 2	R	DF	*P* value
	Inhalation	0.430	645	0.0001
	Major outcome	0.266	645	0.0001
	Chest pain	0.507	645	0.0001
	History of drug abuse	0.245	645	0.0001
	Age	0.227	645	0.0001
Cocaine poisoning	History of illicit drug abuse	0.283	645	0.0001
	Unresponsiveness	−0.234	645	0.0001
	Hospital admission	0.181	645	0.0001
	Antidep antipsych	0.173	645	0.0001
	Prescription medications	−0.353	645	0.0001

Predictors for poisoning outcome, asthma, and for the risk of suicide in our population were analyzed using regression analysis. The final predictive stepwise regression model for the final poisoning outcome was determined as:

Final outcome of poisoning = 0.108 + 0.200 * Cocaine + 0.114 * Inhalation + 0.121 * CVD + 0.103 * Asthma + 0.94 * Gender + 0. 09 * Prior poisoning history

Cocaine was the most predictive variable for having a serious, major final outcome of poisoning, followed by cardiovascular disease, route of inhalation, asthma, gender, and prior history of poisoning.

Stepwise regression was performed to detect significant predictors of asthma cases amongst those admitted to the ED for poisoning. The final predictive stepwise regression model was determined as:

Asthma = 0.419 + 0.221 * Inhalation + 0.147 * Prior poisoning history − 0.140 * Gender + 0.149 * Heroin + 0.728 * Cancer + 0.136 * Race_AA − 0.004 * Age

Surprisingly, a history of cancer was the greatest predictive variable for asthma in the current dataset. Inhalation was the second strongest predictive variable followed by heroin use, prior poisoning history, gender, African American race, and age.

Stepwise regression was performed to determine predictive variables for heroin exposure, in particular, to examine whether or not asthma may predispose patients to abuse heroin. The final predictive stepwise regression model was determined as:

Heroin = 0.284 + 0.794 * Rectal/Vaginal route + 0.142 * Asthma + 0.273 * Inhalation + 0.394 * Injection − 0.193 * CVD − 0.110 * Prior documented poisoning history − 0.077 * Alcohol + 0.111 * Psychosis

Rectal/vaginal and injection routes of exposure were the two most predictive variables for heroin use given the fact that they were the routes of administration the most commonly seen in the population studied. However, inhalational exposure and asthma were also strong predictors, followed by a history of cardiovascular disease, psychosis, prior poisoning history, and alcohol use.

Poisoning and drug addiction remain a significant burden on the healthcare system. The mean LOS for asthmatics without drug abuse was 1.21 days. For asthmatic patients with a history of illicit drug poisoning the mean LOS was 7.07 days. The hospital charges ranged between $99.00 and $18,096.00 with a mean of $2,941 for asthmatics without abuse and between $52.00 and $695,874.00 with a mean of $28,028.00 for the group of asthmatics with illicit drug poisoning. The difference between the LOS for patients with asthma who abused heroin and/or cocaine compared to LOS of asthmatics without abuse was statistically significant (*P* < 0.001), as was hospital cost (*P* < 0.0001.).

## DISCUSSION

Poisoning in the ED is an ever-present issue, and recreational drug related poisoning remains a particular concern for emergency health care providers. In exploring the relationship between asthma and heroin, our data revealed that there are two other significantly correlated co-morbidities with drug abuse, particularly chronic hypertension and cardiovascular diseases that are correlated to cocaine use. As well, the predictive regression model for asthma indicated a potentially strong relationship between asthma and a history of cancer.

The relationship between asthma and drug abuse may be complex, but the observed correlation in this study provides evidence that asthma may be a predisposing factor for heroin addiction and that asthma may be exacerbated by heroin abuse. Several cases have been reported in recent years that suggest sudden and severe asthmatic responses can be temporally correlated to the abuse of cocaine and heroin, such as mortality reports in the state of Maryland that demonstrated a large percentage of patients who died from asthma had positive post mortem urine drug tests.[12] It is unlikely that this results from a receptor mediated effect on the bronchus as opiates favor blocking bronchoconstriction through these receptors rather than stimulating it. It has been suggested that the effect of heroin on bronchoconstriction might occur through a non-receptor mediated mechanism that may be potentiated by an inflammatory response. No supporting evidence has been produced regarding this theory other than the implication that heroin may deregulate mast cells and induce histamine production.

Asthma patients it seems, tend to use various drugs for abuse, both legal and illicit, such as illegal inhalers and prescription drugs (including those that are not widely abused i.e., glucorticoids and salbutamol).[13–15] One study found that high school students with current asthma used cigarettes, cigars, marijuana, and inhalants (huffing) at rates greater than high school students without current asthma.[16,17] It may be that asthmatics experience higher anxiety, which might encourage a patient to reduce their level of anxiety by abusing various drugs.

Whereas the mechanism of action for heroin use on asthma remains poorly characterized, the correlation between cocaine use and cardiovascular disease might be more readily explained due to the effect of cocaine on the heart and blood vessels. Cocaine has also been shown to have numerous other effects on cell signaling systems and the vascular bed by modifying peripheral resistance and inducing a potent vasospasm that raises the blood pressure and increases the cardiac output.[18] By the same mechanism, cocaine induces a vasoconstriction in coronary vessels, which can result in chest pain that mimics angina or a myocardial infarct. Cocaine abusers may present with chest pain similar to angina or coronary infarct that could result in hospitalization. On discharge, a patient will generally be categorized as having a history of chest pain that will be documented in the patient's medical history but without any mention of its temporal relationship to cocaine.

There are several potential limitations to this study. The study is retrospective and the information was collected from the patients' charts, which varied in accuracy and availability between cases. Due to the high rates of drug abuse history, it does not seem that this study underestimated the abuse; however, it might have underestimated some co-morbidity such as arthritis, and particularly depression, which is still not screened systematically for every patient visiting ED, especially in a setting of drug overdose emergencies. Another potential limitation of this study was that there was no way for the study to follow up on specific cases and screen patients for suicidal ideation to find out if they have had any intent for self-harm. Future studies might be designed to avoid these limitations by systematically screening of suicidal intent in these poisoning cases as well as assessing undiagnosed depression and other preexisting mental disorders.

## CONCLUSION

The results of this study provides supporting evidence that deliberate poisoning with illicit drugs remains a serious healthcare issue that significantly aggravates co-morbidities and raises treatment costs by increasing both the rate of hospitalization and hospital length of stay. Heroin abuse is a burden on asthma patients in terms of both their health and hospital costs, particularly for those that are not covered by insurance. Asthma education and self-management techniques that acknowledge the impact of the use of recreational drugs, specifically heroin or cocaine by insufflations, needs to be provided and patients must be educated on how to prevent significant asthma exacerbation by avoiding common triggers, drug use, and other additional stressors.
